# Supplementary material for: Impact of Newborn Screening and Early Dietary Management on Clinical Outcome of Patients with Long Chain 3-Hydroxyacyl-CoA Dehydrogenase Deficiency and Medium Chain Acyl-CoA Dehydrogenase Deficiency—A Retrospective Nationwide Study
Source: Nutrients. 2021 Aug 24;13(9):2925. doi: 10.3390/nu13092925 (PMC8469775; doi:10.3390/nu13092925)
Supplement: Supplementary file 1 [file nutrients-13-02925-s001.zip › nutrients-1342379-supplementary.pdf]

Symptom	Definition	Score
**Acute episodes**		
**Acute encephalopathy**		
mild	somnolence	1
moderate	stupor	2
severe	coma	3
**Rhabdomyolysis**		
mild	CK 660-1500 IU/L (11-22 μkat/L)	1
moderate	CK 1501-10,000 IU/L (22-170 μkat/L)	2
severe	CK 10,001-40,000 IU/L (170-680 μkat/L)	3
critical	CK >40,000 IU/L (>680 μkat/L)	4
**Chronic complications**		
**Retinopathy**		
mild	Hypopigmentation and pigment clumping particularly in the macula; normal vision	1
moderate	Progressive chorioretinal atrophy in the posterior pole, relative sparing of the central macula; paracentral scotoma, progressive myopia, deteriorated colour vision	2
severe	Total atrophy of the posterior pole, posterior staphyloma, sparing of the peripheral fundus; central scotoma	3
**Peripheral neuropathy**		
mild	Decrease/absence of tendon reflexes, no functional limitation	1
moderate	Gait abnormalities, no need for orthoses/other orthopaedic interventions	2
severe	Significant limitation of walking distance and foot deformities necessitating use of orthoses, wheelchair or surgical interventions	3
**Intellectual impairment**		
borderline	IQ 70-79	0.5
mild	IQ 50-69	1
moderate	IQ 35-49	2
severe	IQ 20-34	3
profound	IQ<20	4

Table S1. Severity score definitions for LCHADD/MTPD

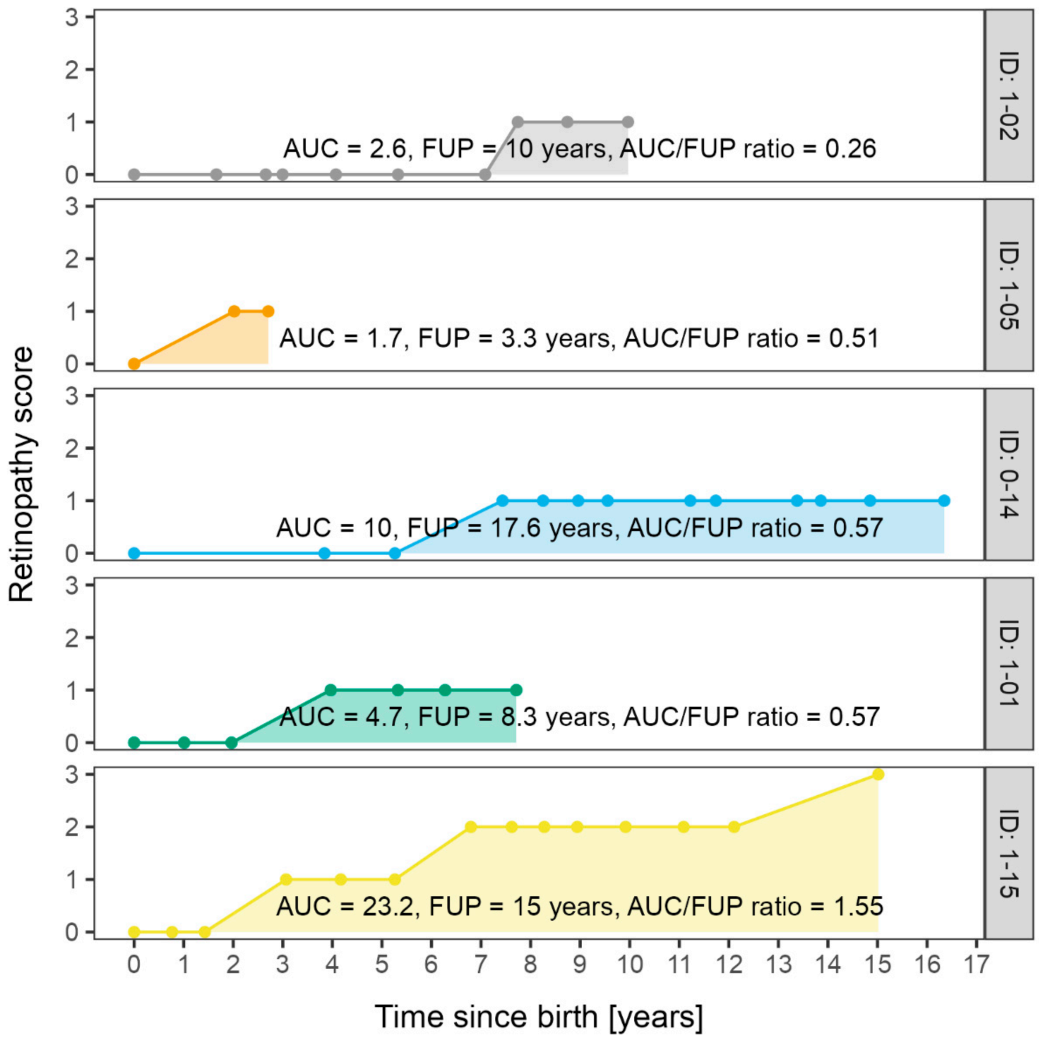

Figure S1: Illustration of severity score calculation in five patients affected by retinopathy. The AUC/FUP ratio reflects the severity of retinopathy as it combines severity score at a given moment together with time of follow-up. We can see that retinopathy in patient 1-15 was approximately 3 times more severe than in patient 0-14. AUC= area under curve; FUP= follow-up period.

ACADM				
Allele 1	Predicted effect	Allele 2	Predicted effect	Patients (N)
c.985A>G	p.Lys329Glu	c.31-1323_118+923del	(deletion of exon 2), r.31_118del88	1
c.985A>G	p.Lys329Glu	c.134A>G	p.Gln45Arg	1
c.985A>G	p.Lys329Glu	c.199T>C	p.Tyr67His	5
c.985A>G	p.Lys329Glu	c.346T>G	p.Cys116Gly	1
c.985A>G	p.Lys329Glu	c.347G>A	p.Cys116Tyr	2
c.985A>G	p.Lys329Glu	c.387+1delG	missplicing	3
c.985A>G	p.Lys329Glu	c.449_452delCTGA	p.Thr150ArgfsTer4	1
c.985A>G	p.Lys329Glu	c.475delT	p.Cys159ValfsTer1	1
c.985A>G	p.Lys329Glu	c.614C>T	p.Ala205Val	1
c.985A>G	p.Lys329Glu	c.616C>T	p.Arg206Cys	3
c.985A>G	p.Lys329Glu	c.727C>T	p.Arg243Ter	1
c.985A>G	p.Lys329Glu	c.985A>G	p.Lys329Glu	43
c.199T>C	p.Tyr67His	c.387G>A	missplicing (deletion of exon 5), r.286_387del101	1
c.347G>A	p.Cys116Tyr	c.347G>A	p.Cys116Tyr	1
c.347G>A	p.Cys116Tyr	c.387G>A	missplicing (deletion of exon 5), r.286_387del101	1
c.347G>A	p.Cys116Tyr	c.421delC	p.Gln141LysfsTer8	1
c.347G>A	p.Cys116Tyr	c.734C>T	p.Ser245Leu	1
c.1114dupG	p.Gly372AlafsTer11	c.(945+1_946-1)_(*1_?)del (deletion of exons 11-12)	nonsense mediated decay?	1
HADHA				
c.1528G>C	p.Glu510Gln	c. (?_-1)_(975+1_976-1)del (deletion of exons 1-10)	nonsense mediated decay?	1
c.1528G>C	p.Glu510Gln	c.58delC	p.Arg20AlafsTer17	1
c.1528G>C	p.Glu510Gln	c.67+2986_315-848del	(deletion of exons 2-4), r.68_314del247	2
c.1528G>C	p.Glu510Gln	c.274_278delTCATC	p.Ser92LysfsTer10	1
c.1528G>C	p.Glu510Gln	c.278C>G	p.Ser93Ter	1
c.1528G>C	p.Glu510Gln	c.703C>T	p.Arg235Trp	1
c.1528G>C	p.Glu510Gln	c.799+5_799+17del	missplicing (deletion of exon 8), r.677_799del123	1
c.1528G>C	p.Glu510Gln	c.914T>A	p.Ile305Asn	1
c.1528G>C	p.Glu510Gln	c.1528G>C	p.Glu510Gln	15
c.1528G>C	p.Glu510Gln	c.1646G>C	p.Arg549Thr	1
c.703C>T	p.Arg235Trp	c.703C>T	p.Arg235Trp	1
HADHB				
c.739C>T	p.Arg247Cys	c.(1389+1_1390-1)_(*1_?)del (deletion of about 6700 bp -	nonsense mediated decay?	1

		from exon 16 to 3'UTR)		
c.1091A>G	p.Glu364Gly	c.1282G>T	p.Gly450Cys	1

Table S2. Genetic variants detected in the *ACADM* and *HADHA*/*HADHB* genes in patients diagnosed with MCADD and LCHADD/MTPD respectively.
